# Fitness to plead: Development and validation of a standardised assessment instrument

**DOI:** 10.1371/journal.pone.0194332

**Published:** 2018-04-26

**Authors:** Penelope Brown, Daniel Stahl, Elizabeth Appiah-Kusi, Rebecca Brewer, Michael Watts, Jill Peay, Nigel Blackwood

**Affiliations:** 1 Department of Forensic and Neurodevelopmental Sciences, Institute of Psychiatry, Psychology and Neuroscience, King’s College London, London, United Kingdom; 2 Department of Biostatistics, Institute of Psychiatry, Psychology and Neuroscience, King’s College London, London, United Kingdom; 3 Department of Clinical, Educational and Health Psychology, University College London, London, United Kingdom; 4 Department of Law, London School of Economics, London, United Kingdom; Grand Valley State University, UNITED STATES

## Abstract

The ability of an individual to participate in courtroom proceedings is assessed by clinicians using legal ‘fitness to plead’ criteria. Findings of ‘unfitness’ are so rare that there is considerable professional unease concerning the utility of the current subjective assessment process. As a result, mentally disordered defendants may be subjected unfairly to criminal trials. The Law Commission in England and Wales has proposed legal reform, as well as the utilisation of a defined psychiatric instrument to assist in fitness to plead assessments. Similar legal reforms are occurring in other jurisdictions. Our objective was to produce and validate a standardised assessment instrument of fitness to plead employing a filmed vignette of criminal proceedings. The instrument was developed in consultation with legal and clinical professionals, and was refined using standard item reduction methods in two initial rounds of testing (n = 212). The factorial structure, test-retest reliability and convergent validity of the resultant instrument were assessed in a further round (n = 160). As a result of this iterative process a 25-item scale was produced, with an underlying two-factor structure representing the foundational and decision-making abilities underpinning fitness to plead. The sub-scales demonstrate good internal consistency (factor 1: 0·76; factor 2: 0·65) and test-retest stability (0·7) as well as excellent convergent validity with scores of intelligence, executive function and mentalising abilities (p≤0·01 in all domains). Overall the standardised Fitness to Plead Assessment instrument has good psychometric properties. It has the potential to ensure that the significant numbers of mentally ill and cognitively impaired individuals who face trial are objectively assessed, and the courtroom process critically informed.

## Introduction

Significant numbers of mentally ill and cognitively impaired individuals pass through the criminal justice system every week. In a proportion of these cases, psychiatrists and psychologists may be asked to determine whether the defendants are capable of fairly standing trial. Clinical assessments of the defendant’s ‘fitness to plead’[1](FTP) are used by the judge to determine whether the trial should go ahead. In England and Wales, this determination rests on professional application and interpretation of case law concerning the Pritchard Criteria (*R v Pritchard* (1836)), more recently outlined in *M (John)* ([2003] EWCA Crim 3452). In order to be fit to plead, a defendant must understand the charge(s), decide whether to plead guilty or not, exercise the right to challenge jurors, instruct solicitors or advocates, follow the course of proceedings and give evidence in his or her own defence. Without one or more of these abilities, a conventional trial should not proceed (*R v Podola*, 1960). Such ‘unfit’ defendants are typically diverted away from their criminal trial to be treated by psychiatric or social services. However, only a very small number of defendants are found unfit to plead in England and Wales (around one hundred[2] out of around 86,000 Crown Court defendants (0.1%) per year). This figure is particularly striking given the extent of cognitive impairment and mental illness in the defendant population. There is therefore considerable professional concern that the procedure for identifying unfit defendants in England and Wales is not fit for purpose[3–5]. The Pritchard Criteria do not consider the decision-making capacity and autonomy of the individual, which are of central importance in contemporary clinical practice and in civil proceedings. They are also not clearly aligned with the test for “effective participation” in criminal trials (*SC v United Kingdom* (2005)) which underpins Article 6 of the European Convention on Human Rights (ECHR)–the right to a fair trial[6]. Clinicians are inconsistent in applying the legal criteria in their assessments, often using arbitrary or no criteria to come to a recommendation[3, 7–9], thus rendering the clinical assessment of FTP unreliable. As a result of these significant concerns the Law Commission of England and Wales published two consultation papers on FTP[10]^,^[11], which proposed legal and procedural reforms. They recommended that a defined psychiatric test to assist in the assessment of FTP should be developed and that the Pritchard test should be replaced by a new legal standard. In their final report published in 2016[12] the Law Commission propose a statutory test of “Capacity to Participate Effectively” (set out in draft legislation in the Criminal Procedure (Lack of Capacity) Bill[13]) to replace the Pritchard Criteria in order to determine FTP. This test essentially has two components. The first incorporates many of the abilities contained within the existing Pritchard criteria, as well as any other ability that appears to the court to be relevant in the particular case. The second is a test of decision-making capacity akin to the civil test for mental capacity contained within the Mental Capacity Act 2005. Similar reforms have already been made in Jersey (Channel Islands)[14] and Scotland[15], and have been proposed in Australia, where various States are also considering incorporating decision-making capacity into the legal test for FTP[16]. Revision of the legal test alone is unlikely to improve the validity of clinical assessments of fitness to plead or to improve clinicians’ abilities to relate clinical findings to the relevant legal criteria- a well-established flaw in forensic mental health assessment[17]. A greater understanding of the cognitive and psychological factors underlying fitness to plead is required to assist clinicians in their application of any legal test. Standardisation of the clinical assessment appears central to improving accuracy[1, 3, 18, 19].

There has been little research exploring the relevant cognitive abilities underpinning FTP in the general defendant population beyond an initial study into the relationship between psychiatric symptoms and the Pritchard Criteria[20]. It has been suggested that FTP assessments require a more scientific approach and that standardised assessment, including the use of instruments, should be used as adjuncts to improve accuracy in court findings of fitness to plead[18, 20]. The use of standardised measures of fitness (or competency to stand trial) is commonplace in North America both in clinical practice and in research[21–23]. No such instrument has been developed specifically to assess FTP in England and Wales beyond a preliminary evaluation of a modified American tool (the MacArthur Competence Assessment tool-Fitness to Plead[24]). This however is not widely available to clinicians or researchers. Grisso has advanced a conceptual model for the development and use of assessment instruments for FTP which is widely accepted in North America[25]. Firstly, the instrument should address the specific legal competencies required by the courts (rather than merely the clinical characteristics of the defendant). Secondly, it should have standardised and quantitative procedures for acquiring relevant data. When properly designed and validated, such forensic assessment instruments ensure that the legal standards of fitness are adequately addressed, reduce errors and bias, and provide reliable, valid and reproducible outcomes[25]. This paper describes the initial development and validation of the first standardised assessment instrument for FTP specifically designed to be used in England and Wales. The instrument uses a filmed vignette of scripted court proceedings in order to assess the abilities required to be fit to plead as well as the decision-making abilities of the defendant, and was developed using rigorous psychometric methods of scale development as recommended by Streiner and Norman[26].

## Method

### Initial instrument development: Item generation and court vignette production

Preliminary work was conducted at a meeting of experts convened by the Law Commission. We carried out a systematic review of the existing instruments for assessing fitness in North America and the construct of fitness as currently determined in England and Wales[1], focusing on the key traits to be measured, namely the ability to follow evidence and court proceedings. The construct definition was reviewed by psychiatrists, psychologists, legal academics, legal practitioners and interested lay persons. A list of potential items felt to address the construct was drawn up[27]. We then developed an ecologically valid twenty minute filmed representation of typical Crown Court proceedings. An excerpt based on trial material concerning a case of actual bodily harm (unlawful wounding) was scripted by four experienced criminal barristers together with the research group and then filmed using actors in Southwark Crown Court. The filmed material included point-of-view discussions of case details with the defence team before entering the court, establishing shots of the courtroom structure, a typical exchange between a key witness and the prosecution barrister, a brief period of cross-examination of this witness by a defence barrister, a discussion during a break in the proceedings with the defence barrister concerning case progress and the decision to give evidence, and final questions from the judge concerning the defendant’s decision to give evidence (or not). Questions addressing generic courtroom knowledge and assessing comprehension of the filmed vignette were scripted. Two near identical versions of the film were produced, one for male participants (references to ‘he’ throughout the case material) and one for female participants (references to ‘she’ throughout the case material).The film and the initial scale items and scoring guide were reviewed by legal, psychiatric and psychological experts for content and face validity. The reviewers also checked that the initial list of items was comprehensive. This intentionally led to a relatively lengthy scale that would then be carefully examined empirically to determine which items should be eliminated, modified or retained. The drafted scale items were subjected to QUAID analysis[28] (“Question Understanding Aid”, http://quaid.cohmetrix.com) to ensure their comprehensibility.

### Scale development (alpha testing phase)

The recruited participants were asked to imagine that they were a defendant (“Sam”) appearing in a Crown Court trial. They were given a brief outline of the charge and key prosecution evidence against Sam. Subjects were asked to recount what they had understood about the charge, and once their adequate understanding was ensured, they proceeded to watch the court case film. The film was paused at scheduled points and the participants were asked questions about the excerpt using the scale. These questions assessed case and plea comprehension (understanding of the charge, comprehension of the distinction between a plea of guilty or not guilty), evidence comprehension (factual memory of evidence including errors or disagreement therein and probing of the ability to explain why statements were in error or disagreement) and other aspects of the trial process (understanding of the roles of court personnel and processes). The initial 42-item scale was administered and refined to produce a 29-item scale using standard item reduction methods[29, 30] in two iterative rounds of testing (round 1, n = 100, round 2, n = 112) (see supplemental data for details).

### Scale evaluation (beta testing phase)

The 29-item FTP Assessment (FTPA) scale was tested on a further 160 participants and underwent factor analysis to confirm the proposed 2-factor structure. We then examined the scale for concurrent validity in a final round of testing. As there is no current ‘gold standard’ measure in the area, we were unable to simply compare the new measure with an existing criterion measure. We were however able to explore other aspects of concurrent validity, namely convergent validity (predicted correlations with cognitive function measures such as full scale IQ (FSIQ)) and known group validity (by comparing differences as predicted between groups with predicted high levels of the trait-normal subjects—and groups with low levels of the trait- learning disabled and lower IQ subjects). Internal consistency and reliability were also assessed.

### Participants

The sample included 160 men and women with English as their first language, aged 18–81 years (mean 45.7 years, s.d. 18.3). The sample was stratified to ensure approximately equal numbers of subjects in each of three ability bands (‘below average’,including ‘low average’, ‘borderline’ and ‘extremely low’ IQs, ‘average’ and ‘above average’) as determined by Wechsler Adult Intelligent Scale–Fourth Edition (WAIS-IV) FSIQ scores of 59–89, 90–109 and 110 and above respectively., The sample was additionally balanced so as to have approximately equal numbers of men and women in each of these bands from each of the four age groups 18–31, 32–47, 48–63 and 64–81. Participants had no self-reported life time history of major mental disorder symptomatology (feeling very low in spirits, feeling very high and overly elated or having had experiences which are difficult to explain, such as hearing voices or seeing things) or substance misuse problems (having problems due to alcohol or other substances) as assessed by the screening questions from the Schedules for Clinical Assessment in Neuropsychiatry (SCAN)[31]. Researchers were trained in assessing capacity to consent to research according to the Mental Capacity Act (2005). Participants lacking capacity to consent and those who were unable to understand the instructions required to undergo the FTPA (for example due to moderate or severe learning disability) were excluded. Participants had no self-reported history of prior criminal convictions or cautions. A subset of 24 participants underwent a second assessment using the FTPA within four weeks of initial assessment to examine test-retest reliability.

### Procedures

This study was approved by the Psychiatry, Nursing and Midwifery Research Ethics Subcommittee at Kings College London (reference PNM/08/09-77) for testing the normal intelligence subjects and by the NRES Committee London for testing in the mild learning disability group of subjects (reference 10/H0807/53). Participants were recruited through local community websites and local community learning disability services and paid minimum hourly wage for their time. Following completion of informed consent, participants completed two sessions of research assessments, one lasting about 45 minutes (demographics and the FTPA instrument), and one lasting about 90 minutes (psychometric instruments). Assessments for this part of the study were conducted between January and December 2012. The clinical raters (EAK and PB) had either masters level training in clinical psychology or post-doctoral level training in clinical forensic psychiatry and had received further training in psychometric assessments from an experienced doctoral-level clinical psychologist (MW).

### Measures

All participants completed the FTPA, the WAIS-IV and the Wechsler Memory Scale (Fourth Edition) (WMS-IV) auditory verbal memory index items. Their executive function was further assessed using the Hayling and Brixton tests[32] and their mentalising abilities by the Theory of Mind- Stories test[33], a set of six short stories used to examine participants’ ability to understand states of false belief (three stories) and of another’s intention to deceive (three stories).

### Statistical analysis

Statistical analysis was carried out using Stata 13. We carried out iterated principal factor analysis, using scree plot, eigenvalues (>1), factor loadings (≥0.3), item cross-over (loadings ≥0.3 on more than one factor), equivalent loadings (loadings on more than one factor within 0.2) and factor content to determine the underlying factor structure. Internal consistency of the scale and individual factors was assessed using Cronbach’s α and test-retest reliability using intraclass correlation coefficients. Pearson’s correlation coefficients were used to examine concurrent validity of the FTPA with measures of IQ, memory, executive function and mentalising abilities.

## Results

### Participants

See Table 1 for details of participant characteristics.

**Table 1:** Respondent characteristics (validation sample) n = 160.

Characteristic	n (%)
**Gender**	
Male	84 (52.5)
Female	76 (47.5)
**Age Group**	
18–31	42 (26.3)
32–47	42 (26.3)
48–63	39 (24.4)
64–81	37 (21.1)
**Ethnicity**	
White	115 (71.9)
Black	26 (16.3)
Asian	12 (7.5)
Other/unknown	7 (4.4)
**Number of Previous Court Attendances**	
0	72 (45.5)
1–3	64 (40.5)
4+	22 (14)
**Full Scale IQ Group**	
Low (59–89)	50 (31.3)
Average (90–109)	60 (37.5)
High (110–150)	50 (31.3)

### Factor analysis

We used an iterated principal factor analysis using oblique rotation to examine the underlying factor structure of the FTPA which confirmed the two-factor solution (Table 2). The two factors showed only a small correlation as expected (r = 0.2). The first factor (71.5% of the variance) contained fourteen items with rotated factor loadings above 0.3. One further item (item 14, addressing the ability to follow trial evidence) had a rotated factor loading of 0.26 but was retained as it was deemed to have theoretical significance to the scale. Items 8, 9 and 26 were not retained due to inadequate loading onto either factor. Item 27 had a rotated factor loading of 0.36 on factor 1 and only 0.16 on factor 2, but was not retained as it is closely linked with item 26 and has a poor theoretical fit on the first factor. The fifteen items retained in the first factor represent the foundational abilities required at court, such as understanding the charge, evidence and pleas, the roles of the court personnel, and the ability to follow the trial. This factor is hereafter referred to as the Foundational Abilities (FA) subscale.

**Table 2:** Rotated factor matrix of the 29 item FTPA-instrument.

Item	Factor 1 (FA)	Factor 2 (DMA)
1. Recall of vignette	**0.60**	0.08
2. Understanding of charge	**0.52**	-0.01
3. Understanding of “not guilty”	**0.36**	0.14
4. Understanding of “guilty”	**0.43**	0.22
5. Understanding of evidence	**0.46**	-0.11
6. Role of Judge	**0.45**	0.08
7. Role of Defence Barrister	**0.66**	-0.14
8. *(Should defence always act in client’s best interests*?	*-0*.*03*^*i*^	*0*.*06)*
9. *(Should defence always follow client’s instructions*?	*-0*.*12*^*i*^	*-0*.*02)*
10. Role of Prosecution Barrister	**0.59**	0.02
11. Role of jury	**0.47**	0.05
12. Role of defendant	**0.35**	-0.06
13. Understanding consequences of emerging evidence	**0.31**	0.12
14. Following trial evidence (group issue)	0.26^ii^	0.20
15. Following trial evidence (leaving the scene)	**0.39**	0.00
16. Understand advantages of giving evidence as a defendant	**0.30**	0.00
17. Understand disadvantages of giving evidence as a defendant	**0.58**	0.05
18. Appreciation of progress of case	-0.41	**0.38**
19. Reasoning abilities concerning case progression	0.09	**0.36**
20. Appreciation of fair treatment in the case	-0.20	**0.52**
21. Reasoning abilities concerning fair treatment	0.03	**0.45**
22. Appreciation of possible case outcomes (guilty or not)	0.05	**0.34**
23. Reasoning abilities concerning case outcomes	0.14	**0.37**
24. Appreciation of how guilty verdict will affect life	-0.07	0.29^ii^
25. Reasoning concerning impact of guilty outcome	0.25	0.23^ii^
26. *(Appreciation of how not-guilty outcome will affect life*	*0*.*13*	*-0*.*01*^*i*^*)*
27. *(Reasoning regarding impact of not-guilty outcome*	***0*.*36***^***iii***^	*0*.*16)*
28. Understanding and appreciation of possible penalties in the event of a finding of guilt	0.26	**0.39**
29. Reasoning abilities concerning possible penalties	**0.33**	**0.46**

Italicised Items (8,9,26,27) were not retained in the final instrument

i. factor loading <0.3, item not retained

ii. factor loading <0.3 but item retained

iii. factor loading >0.3 but item not retained

FA = Foundational abilities DMA = Decision-making abilities

The second factor (36% of the variance) contained eight items with rotated factor loadings above 0.3. One additional item (item 24) had a factor loading of 0.29 and was also retained. One further item (item 25) had a factor loading of 0.23 on the second factor subscale and 0.25 on the first factor subscale. We elected to retain this item on the factor 2 subscale as it is closely linked to item 24 and has a better theoretical fit within the second subscale. The items in this subscale assess decision-making abilities; in particular how well participants could appreciate the progress of the case and its impact on their lives as well as their reasoning abilities, and is hereafter referred to as the decision-making abilities (DMA) subscale. Item 29 has loadings of >0.3 on both factors but was retained in the DMA subscale as the factor loading was higher for this subscale and it has a better theoretical fit with DMA. Overall, ten items were retained in the DMA subscale.

The pattern of score distributions for the final 25-item instrument and the two subscales is shown in Table 3.

**Table 3:** Score distributions and reliability of the FTPA-instrument and sub-scales.

	FTPA (total)	FA subscale (factor 1)	DMA subscale (factor 2)
**Mean score (s.d.)**	**42.0 (7.9)**	**21.3 (5.3)**	**20.8 (4.1)**
**Correlations (Pearson’s r)**			
- with age	-0.09	-0.01	-0.06
- with gender	0.02	-0.01	0.04
- with number of court appearances	0.12	0.16*	0.03
**Reliability**			
Cronbach’s α	N/A	0.76**	0.65
Test-retest (icc)	0.70**	0.83**	0.32

* p<0.05

**exceeds acceptable level of 0.7

FTPA = Fitness to Plead Assessment Instrument

FA = Foundational abilities

DMA = Decision-making abilities

### Reliability

Test-retest reliability and internal consistency analyses are presented in Table 3. No single item deletion improved the internal consistency to above 0.80. Item-total correlations exceeded 0.3 for all individual items apart from item 8 (0.13) and item 18 (0.15).

### Validity

Table 3 shows no significant correlations between the FTPA total and subscale scores and gender and age suggesting that scores on the instrument are not meaningfully affected by age and gender and supporting the discriminant validity of the instrument and sub-scales. A significant correlation was observed between the number of court appearances by participants and scores on the FA subscale, but not the total score or DMA subscale score.

Table 4 shows the distribution of scores on the tests for cognitive and executive functioning and the correlations between these tests and the FTPA-total and subscale scores. All correlations were statistically significant suggesting excellent convergent validity. Total scores on the FTPA-instrument were highly correlated with tests of cognitive and executive function.

**Table 4:** Score distributions and pearson’s correlations of cognitive and executive function tests with FTPA-instrument total and subscale scores.

	Score Mean (s.d.)	FTPA total r	FA subscale (factor 1) r	DMA subscale (factor 2) r
**WAIS Full Scale IQ**	98.3 (18.69)	0.65***	0.70***	0.34***
**WMS AMI**	96.2 (17.2)	0.52***	0.53***	0.31***
**Hayling Task**	5.4 (1.9)	0.35***	0.34***	0.21*
**Brixton Task**	5.5 (2.3)	0.51***	0.51***	0.28**
**Theory of Mind Test**	43.5 (5.4)	0.51***	0.56***	0.24*

*p≤0.01

**p<0.001

***p<0.0001

WAIS FSIQ = Wechsler Adult Intelligence Scale (4th Edition) Full Scale IQ

WMS AMI = Wechsler Memory Scale (4th Edition) Auditory Memory Index

FTPA = Fitness to Plead Assessment Instrument

FA = Foundational abilities

DMA = Decision-making abilities

## Discussion

### Summary of findings

We have developed and validated the first standardised assessment instrument for FTP specifically designed to be used in England and Wales and other jurisdictions with similar legal standards of fitness. The instrument is a structured interview with prompts and follow-up queries provided for discrete items, with clear anchors for ratings. After a short period of training for the assessor, it is easy and practical to administer in outpatient settings to individuals with a wide-range of intellectual functioning (with FSIQs ranging from 59–150). The final 25-item version takes approximately 35 minutes to administer. The instrument has been validated in subjects with an IQ of greater than 59 because we found that those with lower FSIQs were unable to understand the instructions required to follow the filmed vignette and take part in the study. This suggests that there would be serious concerns about a defendant’s fitness to plead in the event of a psychometric finding of a FSIQ of less than 59, although any judgment would need to be made on a case by case basis incorporating the wider clinical material.

### Factor structure of the FTPA

The emergent factor structure of the FTPA instrument reveals two subscales. The first subscale comprises 15 items which reflect the foundational abilities (FA) required for a defendant to be fit to plead, incorporating the Pritchard Criteria. The second subscale comprises 10 items which reflect the defendant’s decision-making abilities, incorporating the principles of the Mental Capacity Act, namely the ability to use and weigh information and appreciate its relevance to the situation at hand. These abilities reflect the current legal standard of FTP according to the Pritchard Criteria, and also take into consideration relevant decision-making capacity and the ability to effectively participate in court, thereby reflecting the new legal test for fitness proposed by the Law Commission. The factorial structure also mirrors that of the legal standard of adjudicative competence in the USA described by Bonnie as “foundational competence” and “decisional competence”[34].

A number of items in the final FTPA did not achieve the required factor loadings for their respective subscales (namely items 14, 24 and 25), possibly due to random sampling errors, but these were retained due to their theoretical significance to the scale. Item 14 addresses a change in the trial evidence which reflects an important item within the Pritchard Criteria (the ability to follow proceedings), and is useful in determining whether an individual can effectively participate. Items 24 and 25 are linked items which address how an individual understands and appreciates the impact of criminal sanctions on their own lives. Again, we hold that this is an important aspect of the decision-making element of being fit to plead. Further evaluation of the scale with a larger sample of subjects will confirm whether these items do in fact fit within the instrument.

Subscale FA showed excellent internal consistency and test-retest reliability, but the α and icc reliability scores for the DMA subscale fell below the generally accepted levels. The slightly weaker internal consistency (α) could be explained by the weaker statistical properties of the DMA subscale. The test-retest reliability was considerably poorer for this subscale, and one possible explanation is that decision-making abilities are not as stable over time as foundational abilities. While this has been shown to be the case in individuals with mild cognitive impairment[35], decision-making capacity has been shown to be relatively stable over time even in those with major mental illnesses[36], and it is unclear why normal subjects should score differently on the DMA scale over a 4-week period. We also had a small sample (n = 24) for test-retest reliability, and further exploration of the subscales with a larger sample is required.

### Construct validity of the FTPA

Age and gender did not affect scores on the instrument. However, the number of times participants had previously appeared in court correlated positively with scores on the FA subscale but not with the DMA subscale or the total score. Court appearances included being a witness, juror and legal professional, and the number of court appearances made by an individual is likely to reflect the participants’ prior knowledge of the court process. Our finding that the number of times someone has been in court does not correlate with the total FTPA score confirms that the FTPA is not purely a test of court knowledge or experience. The correlation between prior experience of being in court and the foundational abilities tested by the instrument is as expected. We would not expect prior court experience to correlate with decision-making abilities and therefore the lack of correlation of court appearances with the DMA subscale is reassuring.

Intelligence and memory have consistently been found to impact on fitness to plead[19, 37]. As expected, performance on the FTPA (both total scores and individual factor scores) correlates highly with measures of cognitive functioning (WAIS-IV and WMS), but not so highly that IQ and memory measures alone or together could be used as proxy measures of an individual’s ability to participate effectively in court proceedings. The FA-subscale correlated more strongly with FSIQ and memory than the DMA subscale which is as expected as items in the latter subscale (such as an appreciation of the extent to which one is being treated fairly in the court case) could be viewed as less “purely” cognitive in nature. This replicates findings from the validation of the MacCAT-CA, a widely-used North American instrument, in which the “understanding” measures were most highly correlated with verbal cognitive functioning[38].

### Strengths and limitations

The FTPA instrument development process involved qualitative research and consultation with legal professionals from the outset. We have also worked closely with the Law Commission to ensure that both the existing criteria for fitness to plead (the Pritchard Criteria) as well as questions concerning decision-making capacity and effective participation (key areas which are included in their recommended reform of the legal test) are included in the final version of the instrument. The instrument has been reviewed by legal professionals to ensure good face validity and it fulfils Grisso’s requirements for structured instrument development.

Throughout each stage of the instrument development we have been careful to ensure that the sample sizes were large enough to produce statistically valid results, and have used a new sample of participants at each stage. Our final sample of 160 participants included equal numbers of males and females, and was designed to have an even spread of FSIQ ranges in order to carry out known-group methods to compare scores on the FTPA with cognitive ability. However, as noted above we used a relatively small sample for test-retest reliability, and further exploration of the subscales with a larger sample, including inter-rater reliability, is needed to confirm the validity of the FTPA instrument.

This initial validation of the FTPA was confined to normal subjects and those with mild learning disabilities who are not criminal defendants, and hence the study population is not a true representation of the target population for the instrument. The initial version of the instrument was comparatively long, and when combined with the psychometric and cognitive tests, the testing time per subject was around 2 hours. At this early stage of the research process it was more important to establish community norms for FTP by examining individuals with differing IQs. The next stage of the study will be to formally assess performance in individuals engaged in the criminal justice process to further validate the instrument. Our instrument has still to be validated with known groups such as those suffering from mental disorders and those previously determined to be unfit. However the very small number of defendants found unfit each year significantly limits research in this population. We intend to test the instrument for its ability to differentiate malingering subjects from those with genuine cognitive impairments or mental illnesses.

The filmed vignette approach has significant ecological validity advantages over written vignettes. It is well established that there are low literacy and high specific reading difficulty rates amongst offenders[39], and by having the information about the court process presented audio-visually, individuals being assessed by the FTPA are not required to read or otherwise process large volumes of text, and are more likely to be able to concentrate on the vignette as they are familiar with watching television. The downside of this is that audio-visual equipment (a laptop or video-monitor) is required to administer the instrument, which could have practical implications in secure settings which restrict access to computer equipment (such as prisons and secure hospitals). However we have had no difficulties to date in administering the instrument for research purposes in a follow-up study in custodial settings including courts and prisons.

### Conclusions

Even with these admitted limitations, we believe that with further development the FTPA has significant objective advantages over the current approach. It is not designed to replace clinical assessment of defendants at court, but to be used as an adjunct to provide a standardised, reliable and valid way of determining whether individuals are able to participate effectively in court proceedings. While it has been designed for use in England and Wales, it is likely to be applicable in other common law jurisdictions with a similar legal standard for FTP. Such an instrument should therefore be welcomed by clinicians, legal professionals, defendants and victims for whom the current process is well-recognised as inadequate.

## Supporting information

S1 File (DOCX)
