# Supplementary material for: Fitness to plead: Development and validation of a standardised assessment instrument
Source: PLoS One. 2018 Apr 26;13(4):e0194332. doi: 10.1371/journal.pone.0194332 (PMC5919482; doi:10.1371/journal.pone.0194332)
Supplement: S1 File — (DOCX) [file pone.0194332.s001.docx]

**Online supplemental data**

**Scale Development**

**Alpha Testing Phase 1**

**Participants** The sample included 100 men and women with English as their first language, aged 18-80 years (mean 37.7 years, s.d. 12.9), stratified to ensure approximately equal numbers of subjects in each of three ability bands (‘below average’, ‘average’ and ‘above average’ as determined by Wechsler Adult Intelligent Scale – Fourth Edition (WAIS-IV) scores of 89 and below, 90-109 and 110 and above respectively), and balanced so as to have approximately equal numbers of men and women in each of these bands from each of the four age groups 18-31, 32-47, 48-63 and 64-81. The participants were recruited using community websites.

Participants had no self-reported life time history of major mental disorder symptomatology (feeling very low in spirits, feeling very high and overly elated or having had experiences which are difficult to explain, such as hearing voices or seeing things) or substance misuse problems (having problems due to alcohol or other substances) as assessed by the screening questions from the SCAN^26^. Participants had no self-reported history of criminal convictions. These self-reported criminal histories were subsequently checked via Police National Computer access. Four individuals were found to have at least one conviction or caution recorded on the PNC and they were thus removed from further analysis.

**Procedures** This study was approved by the Psychiatry, Nursing and Midwifery Research Ethics Subcommittee at Kings College London (reference PNM/08/09-77) for testing the normal intelligence subjects and by the NRES Committee London for testing in the mild learning disability group of subjects (reference 10/H0807/53). Participants were recruited through local community websites and local community learning disability services and paid minimum hourly wage for their time. Following completion of informed consent, participants completed two sessions of research assessments, one lasting about 45 minutes (demographics and the FTP instrument), and one lasting about 45 minutes (psychometric instruments). Assessments for this part of the study were conducted between October 2009 and June 2010. The clinical rater (RB) had masters level training in clinical psychology and had received further training in psychometric assessments from an experienced doctoral-level clinical psychologist (MW).

**Measures** All participants completed the FTPA instrument (supplementary Table 1) and the Wechsler Adult Intelligence Scale (Third Edition).

**Supplementary Table 1 FTPA-alpha one instrument scenes and questions (scoring range; higher scores indicate greater ability)**

Scenes 1 and 2: Discussion of case details with the defence team before entering the court

Item 1: Recall of six key details of assault vignette (0-6)

Item2: Recall of six key details of assault vignette following prompting (0-6)

Item 3: Understanding of charge (0-2)

Item 4: Understanding of a not guilty plea (0-2)

Item 5: Understanding of a guilty plea (0-2)

Item 6: Understanding of evidence (0-2)

Scene 3: Establishing shots of courtroom structure and personnel

Item 7: Role of judge (0-2)

Item 8: Role of defence barrister (0-2)

Item 9: Should defence barrister always act in client’s best interests? (0-4)

Item 10: Should defence barrister always follow client’s instructions? (0-4)

Item 11: Role of prosecution barrister (0-2)

Item 12: Role of jury (0-2)

Item 13: Role of defendant (0-2)

Scene 4 & 5: Examination of complainant by prosecution barrister

Item 14: New piece of evidence emerging in examination-in-chief (0-2)

Item 15: Understanding consequences of emerging evidence (0-1)

Item 16: Act of complainant immediately prior to the incident (0-1)

Item 17: Concerns of complainant regarding group containing assailant (0-2)

Item 18: Complainant account of assault (0-1)

Item 19: Complainant account of assailant’s clothing (0-1)

Item 20: Complainant account of weapon involved (0-2)

Item 21: Whether the complainant had previously mentioned a weapon (0-1)

Scenes 6, 7 and 8: Completion of prosecution barrister examination; defence barrister cross-examination; defence barrister request for a break to enable case discussion with defendant

Item 22: Complainant account of site of assault (0-1)

Item 23: Complainant account of injury (0-1)

Item 24: Complainant account of nature of retaliation (0-1)

Item 25: Complainant account of assailant identification on leaving the scene (0-1)

Item 26: Defence barrister concerns about new evidence (0-2)

Item 27: Defence barrister challenge concerning complainant weapon (0-1)

Item 28: Defence barrister challenge concerning clothing of assailant (0-1)

Item 29: Defence barrister challenge concerning nature of defendant’s actions (0-1)

Item 30: Defence barrister challenge concerning new complainant account of assailant clothing (0-2)

Scenes 9 & 10: Discussion of case progress with defence team in a court break; judge discussion with defence barrister concerning the defendant potentially giving evidence

Item 31: Defendant choice concerning giving evidence (0-2)

Item 32: Necessity or otherwise of giving evidence (0-1)

Item 33: Understand advantages of giving evidence as a defendant (0-2)

Item 34: Understand disadvantages of giving evidence as a defendant (0-2)

Item 35: Appreciation of progress of case (0-4)

Item 36: Reasoning abilities concerning case progression (0-2)

Item 37: Appreciation of fair treatment in case (0-4)

Item 38: Reasoning abilities concerning fair treatment (0-2)

Item 39: Appreciation of possible case outcomes (0-4)

Item 40: Reasoning abilities concerning case outcomes (0-2)

Item 41: Appreciation of possible penalties in the event of a finding of guilt (0-1)

Item 42: Reasoning abilities concerning possible penalties (0-2)

**Data analysis** Statistical analysis was carried out using SPSS version 20. Analyses were conducted to examine the psychometric properties and validity of the FTPA-alpha. Item-level descriptive statistics and subscale-level statistics (internal consistency reliability) were examined. Standard psychometric item reduction analyses of the questionnaire were conducted to identify and retain items with strong psychometric properties. These analyses were guided by a well-defined *a priori* item reduction strategy developed in previous work^24,25^. On the basis of standard psychometric tests and these criteria, items with acceptable psychometric properties were retained and those with poor measurement properties were eliminated to produce a shorter, item-reduced version of the FTPA. Supplementary Table 2 summarises the psychometric tests and criteria we used to guide item reduction.

**Supplementary Table 2. Psychometric Tests and Criteria**

|  | **Psychometric Test/Analysis** | **Criteria for retention** |
| --- | --- | --- |
| **Item level analysis** |  |  |
|  | Missing Data | ≤5% |
|  | Maximum endorsement frequencies (ceiling/floor effects) | ≤80%  >5% |
|  | Item redundancy (inter-item correlations) | ≤0.75 |
|  | Internal consistency (item-total correlations) | ≥0.30 |
| **Scale level**  **analyses** |  |  |
| *Reliability* | Internal consistency (Cronbach’s alpha) | ≥0.70 |

**Results *Item Reduction*** Ten items were removed due to ceiling effects (items 14, 16, 19, 21, 22, 23, 24, 29, 31, 32). Five items were removed due to poor item-total correlations (items 2, 18, 20, 26, 27).

***Item modification*** Item scoring anchors for several items (items 5, 13, 15, 41, 42) were not sufficiently sensitive to variation in participant responses and their scoring ranges were thus increased to improve variance and reduce ceiling and floor effects. This was possible because participant responses were recorded verbatim.

***Item generation*** Four new items were generated to assess the participants’ reasoning about the potential impact on their lives of findings of guilty or not-guilty. These were again reviewed by legal, psychiatric and psychological experts for content and face validity before their introduction into the alpha testing phase 2 version of the instrument.

**Discussion** The results provided initial support for the feasibility and validity of this approach to assessing fitness to plead. These preliminary data were especially encouraging given that this was the first field test of the new instrument and the item content, interview probes and provisional manual were all in the development phase at the time of the study. The internal consistency of the full scale score was encouraging (Cronbach alpha = 0.74). Item-level statistics suggested some areas of concern and led to item reduction of fifteen items, item anchor scoring modification of five items and item generation of four new scale items. This resulted in a thirty one item scale for use in the next round of testing.

**Alpha Testing Phase 2**

**Participants** The sample included 112 men and women with English as their first language, aged 18-80 years (mean years 41.1, s.d. 15.2), stratified, recruited and assessed as per the Alpha Testing Phase 1 procedure. Assessments for this part of the study were conducted between January 2011 and December 2011. The clinical rater (EAK) had masters level training in clinical psychology and had received further training in psychometric assessments from an experienced doctoral-level clinical psychologist (MW). Police National Computer Records were not accessed for this group following further discussion with the PNC authorities concerning the necessity of further records requests given the very low numbers of criminal records observed in alpha testing phase 1.

**Measures** All participants completed the FTPA instrument (alpha two version, Supplementary Table 3) and the Wechsler Adult Intelligence Scale (Fourth Edition).

**Supplementary Table 3 FTPA-alpha two instrument scenes and questions (scoring range; higher scores indicate greater ability)**

Scenes 1 and 2: Discussion of case details with the defence team before entering the court

Item 1: Recall of six key details of assault vignette (0-6)

Item 2: Understanding of charge (0-2)

Item 3: Understanding of a not guilty plea (0-2)

Item 4: Understanding of a guilty plea (0-4)

Item 5: Understanding of evidence (0-2)

Scene 3: Establishing shots of courtroom structure and personnel

Item 6: Role of judge (0-2)

Item 7: Role of defence barrister (0-2)

Item 8: Should defence barrister always act in client’s best interests? (0-4)

Item 9: Should defence barrister always follow client’s instructions? (0-4)

Item 10: Role of prosecution barrister (0-2)

Item 11: Role of jury (0-2)

Item 12: Role of defendant (0-3)

Scene 4 & 5: Examination of complainant by prosecution barrister

Item 13: Understanding consequences of emerging evidence (0-2)

Item 14: Concerns of complainant regarding group containing assailant (0-2)

Scenes 6, 7 and 8: Completion of prosecution barrister examination; defence barrister cross-examination; defence barrister request for a break to enable case discussion with defendant

Item 15: Complainant account of assailant identification on leaving the scene (0-1)

Item 16: Defence barrister challenge concerning clothing of assailant (0-1)

Item 17: Defence barrister challenge concerning new complainant account of assailant clothing (0-2)

Scenes 9 & 10: Discussion of case progress with defence team in a court break; judge discussion with defence barrister concerning the defendant potentially giving evidence

Item 18: Understand advantages of giving evidence as a defendant (0-2)

Item 19: Understand disadvantages of giving evidence as a defendant (0-2)

Item 20: Appreciation of progress of case (0-4)

Item 21: Reasoning abilities concerning case progression (0-2)

Item 22: Appreciation of fair treatment in case (0-4)

Item 23: Reasoning abilities concerning fair treatment (0-2)

Item 24: Appreciation of possible case outcomes (0-4)

Item 25: Reasoning abilities concerning case outcomes (0-2)

Item 26: Appreciation of how guilty outcome will affect life (0-3)

Item 27: Reasoning concerning impact of guilty outcome (0-3)

Item 28: Appreciation of how not-guilty outcome will affect life (0-3)

Item 29: Reasoning concerning impact of not-guilty outcome (0-3)

Item 30: Appreciation of possible penalties in the event of a finding of guilt (0-3)

Item 31: Reasoning abilities concerning possible penalties (0-3)

**Data analysis** Statistical analysis was carried out using SPSS version 20. Analyses were conducted to examine the psychometric properties and validity of the FTPA-alpha two. Item-level descriptive statistics and subscale level statistics were examined.

**Results *Item Reduction*** Two further items were removed due to poor item-total correlations (items 16 and 17)

**Discussion** The results provided further support for the feasibility and validity of this approach to assessing fitness to plead. Two further items were excluded for psychometric reasons, resulting in a twenty nine item instrument for use in the scale evaluation phase of testing.
